# Management of Patients With Opioid Use Disorder (OUD)

**DOI:** 10.7759/cureus.91786

**Published:** 2025-09-07

**Authors:** Nathan Martin-Orr, Steve Yun

**Affiliations:** 1 Anesthesia, University of California Irvine Medical Center, Orange, USA; 2 Anesthesia, Western University of Health Sciences, Pomona, USA

**Keywords:** buprenorphine, methadone, multimodal analgesia, opioid-agonist therapy, opioid-induced hyperalgesia, opioid use disorder, perioperative pain management, regional anesthesia

## Abstract

Managing patients with opioid use disorder (OUD) presents a critical challenge in the perioperative setting. Due to challenges like opioid-induced hyperalgesia (OIH), withdrawal and relapse risks, and altered opioid pharmacodynamics, traditional pain management strategies may be inadequate or even counterproductive. Despite the inconsistency in clinical guidelines and variation in perioperative protocols for OUD patients, mounting evidence indicates that effective analgesia techniques do exist, such as multimodal analgesia, regional anesthesia, opioid agonist therapy (OAT), and non-opioid adjuncts. Evidence suggests that continuing OAT (methadone or buprenorphine) during the perioperative period may improve pain control and reduce relapse risk. Multimodal analgesia techniques decrease opioid usage while increasing pain relief. This review will highlight literature that demonstrates the effectiveness and benefit of these treatments for OUD patients. The complexity of OUD and its impact on perioperative care requires a patient-centered, multidisciplinary approach in order to increase safety and optimize patient outcomes. This review demonstrates the need to address research gaps, reduce the negative stigma surrounding this disorder, and establish standardized clinical guidelines that prioritize multimodal pain management and inclusive OUD treatment strategies.

## Introduction and background

Opioid use disorder (OUD) is characterized as a chronic relapsing disorder leading to significant impairment or distress, represented by criteria such as tolerance, withdrawal, and continued use despite harm [1]. With opioid-related deaths rising, OUD has become a global health crisis; in 2020, an estimated 16 million people worldwide suffered from this disorder [2]. The ongoing opioid epidemic translates into an increased number of surgical patients having a history of opioid use or dependence. These habits contribute to opioid tolerance, opioid-induced hyperalgesia (OIH), and an increased risk of withdrawal and relapse if pain levels are not properly managed. Because of this, anesthesia providers are unable to use traditional opioid-based analgesia and must employ unique pain management techniques to avoid withdrawal and minimize risks [3].

Effective perioperative treatment and pain management in this population are further complicated by physiological and pharmacological factors. Chronic opioid exposure can result in neuroplastic changes that paradoxically increase pain sensitivity, leading to an increased risk of inadequate analgesia and longer hospital stays. Opioid agonist therapies (such as methadone or buprenorphine) must also be considered by providers who are treating OUD patients; abruptly stopping these treatments can cause withdrawal, emotional distress, and a higher likelihood of postoperative relapse [3,4]. Regardless of these challenges, evidence-based approaches that incorporate multimodal anesthesia tactics, including regional anesthesia, non-opioid analgesics, N-Methyl-D-Aspartate (NMDA) receptor antagonists, and multimodal pain strategies, have shown promising potential in improving pain management and reducing opioid consumption.

Despite ongoing research, a widespread lack of standardized protocols and variation in clinical practice remain. For example, many anesthesia providers hesitate to continue opioid agonist therapies during the perioperative period, in contrast to the clinical evidence suggesting these medications can aid in preventing withdrawal and maintaining stability [2]. Additionally, societal stigma and bias of healthcare professionals lead to suboptimal care and poor patient outcomes [5].

The purpose of this review is to compile current evidence on perioperative pain management techniques for patients in order to improve clinical decision-making and reduce perioperative risks for this highly vulnerable population. The main focus will be on the effectiveness of multimodal anesthesia, opioid agonist therapy, OIH, and the role of regional anesthesia in combating the negative perioperative implications associated with this disorder. This review will provide practical recommendations while simultaneously demonstrating the need for further research to bring needed solutions to OUD treatment.

## Review

Pathophysiology of opioid use disorder and opioid-induced hyperalgesia

Patients with OUD and a history of chronic opioid usage undergo significant neurobiological alterations that affect the way they perceive and experience pain. Changes in the neurobiology of these patients lead to opioid-induced hyperalgesia and other symptoms that complicate successful perioperative pain management. Understanding the root of these changes will allow researchers to develop treatments that can properly combat this pain hypersensitivity.

Neurobiology of OUD

At a cellular level, chronic opioid exposure results in a cascade of both structural and functional changes. These neuroadaptive alterations occur in the opioid receptors, neurotransmitter pathways, and pain modulation pathways. The result of these effects is the development of withdrawal, tolerance, and dependence on opioids. 

One of the main opioid receptors affected is the mu-opioid receptor (MOR), which can facilitate analgesia, euphoria, and respiratory depression when activated. In a patient suffering from OUD, it is common for there to be progressive decreases in MOR signaling due to β-arrestin recruitment to the receptor. β-arrestin prevents any further coupling of G-proteins with the receptor, leading to endocytosis of the receptor endosome and eventual receptor internalization [6]. This receptor internalization causes a reduction in the efficacy of opioids in these patients. Along with this desensitization, there is a significant downregulation of the mu-receptors due to chronic exposure and decreased receptor availability. Consequently, these patients require a higher opioid dosage to experience the same analgesic effect, leading to tolerance and a higher risk of overdose. Additionally, constant kappa-opioid receptor (KOR) activation causes further adaptive changes to take place; KORs are responsible for creating feelings of dysphoria and stress responses. Chronic opioid exposure causes the upregulation of dynorphins in the body, a natural neuropeptide that binds to KORs, bringing a cascading effect that leads to a heightened stress response and perception of pain [7]. Aside from KOR activation, dynorphins activate numerous descending pain facilitation pathways that contribute to hyperalgesia [4]. The effects of KOR activation and MOR adaptations help explain the difficulties of perioperative treatment of OUD patients, specifically why higher opioid doses do not always improve pain control and can even worsen hyperalgesia. 

Along with receptor adaptations and tolerance formation, chronic opioid usage leads to dependence and withdrawal symptoms through a variety of different mechanisms. OUD patients face a widespread reprogramming of brain circuits that alters noradrenergic and dopaminergic signaling. Noradrenergic signaling is altered by changes in locus coeruleus activation; when opioids bind to the MOR on locus coeruleus neurons, they decrease signaling in this nucleus, consequently decreasing norepinephrine release. However, when opioids are abruptly stopped, the locus coeruleus is hyperactive, causing a norepinephrine surge which contributes to withdrawal symptoms such as anxiety, sweating, and tachycardia [6,8]. Dopaminergic signaling is dampened due to a reduction in dopamine receptor sensitivity, creating an impaired reward processing system that brings about feelings of depression and opioid cravings [9]. Another system that promotes dependence and worsens withdrawal is the dynorphin-mediated KOR activation cascade that was previously addressed. By amplifying the stress response, KOR activation heightens emotional distress and leads to drug-seeking behavior [7]. In regard to perioperative treatment of these patients, these noradrenergic and dopaminergic signaling effects have significant clinical impacts. Abrupt cessation of opioids will cause a norepinephrine surge, leading to intense withdrawal symptoms and heightened nociception. Additionally, chronic opioid usage will reinforce physiological dependence and withdrawal-driven opioid dependence.

Mechanisms of Opioid-Induced Hyperalgesia

Opioid induced hyperalgesia (OIH) is a paradoxical condition in which prolonged opioid usage increases pain sensitivity rather than diminishing it. Distinct from tolerance (where opioids become less effective due to long-term exposure), OIH heightens pain perception despite increasing opioid dosage.

Chronic opioid usage causes an increase in glutamate release from presynaptic neurons and promotes NMDA receptor sensitization. This heightened baseline level of glutamate in synaptic terminals surges NMDA receptor activity, sending amplified pain signals to the brain [7]. Another result of this NMDA receptor activation is an influx of calcium into the postsynaptic neuron, which can trigger signaling pathways that promote further glutamate release into the synaptic terminal, creating a positive feedback loop where increased neuronal activity further enhances glutamate release. This calcium influx strengthens synaptic plasticity and central sensitization, demonstrating its crucial role in the development of OIH [3]. Another harmful effect of NMDA sensitization is excitotoxicity, where this excessive activity causes damage to these neurons, worsening pain perception further. As a result of these NMDA receptor modulations, increasing opioid doses can worsen OIH and pain levels in OUD patients, requiring opioid-sparing techniques such as NMDA antagonists [2]. Ketamine, an NMDA receptor antagonist, has shown promise in mitigating the effects of OIH and even reversing it [10].

Another harmful effect of opioid usage is the overexpression of spinal dynorphins in the dorsal horn of the spinal cord. Excessive spinal dynorphin presence can have a number of effects on an individual with OUD and further contribute to OIH. By consistently activating KORs, these dynorphins cause neuroinflammation and worsen pain sensitivity through multiple mechanisms [7]. Dynorphin-mediated KOR activation facilitates a signaling pathway that results in downstream production of excitatory neurotransmitters associated with nociception, such as substance P and calcitonin gene-related peptide (CGRP), leading to enhanced pain signaling to the brain from the spinal cord [4]. In addition, elevated dynorphin levels can induce the release of proinflammatory cytokines and chemokines within the spinal cord, creating an inflammatory environment that sensitizes nociceptive pathways and enhances pain perception. Lastly, dynorphins contribute to the previously discussed dysregulation of NMDA receptors and glutamate levels, bolstering the effects of OIH [3]. Ultimately, the elevated levels of spinal dynorphins make pain more diffuse and resistant to the effects of opioids. As a result of the widespread inflammation that results, implementing anti-inflammatory agents, such as cyclooxygenase (COX)-2 inhibitors and dexamethasone, is crucial in counteracting the pain sensitization brought on by dynorphins.

One of the final mechanisms by which OIH manifests in patients is neuroinflammation and central sensitization. Chronic opioid usage stimulates the activation of microglia and astrocytes in the spinal cord, leading to a number of effects. Pro-inflammatory cytokines are released [tumor necrosis factor (TNF)-alpha, IL-1β], causing a direct activation of nociceptors, leading to neuronal excitability and lowered pain thresholds [7]. These inflammatory mediators can further damage the junctions between endothelial cells, increasing permeability of the blood-brain barrier. As a result, inflammatory mediators and signaling molecules from the periphery can easily filter into the central nervous system (CNS), contributing to the central sensitization that characterizes OIH [11]. Ultimately, neuroinflammation worsens both tolerance and hyperalgesia, requiring providers to consider glial inhibitors and anti-inflammatory agents [such as nonsteroidal anti-inflammatory drugs (NSAIDS) or COX-2 inhibitors] to counteract opioid-induced neuroinflammation and pain amplification [10]. 

Pain Perception Alterations and Implications for Perioperative Pain Control

The neurobiological changes that take place in OUD patients result in abnormal pain processing, heavily influencing perioperative pain management. Typical opioid analgesia levels are blunted by higher tolerance and OIH [12]. Along with decreased analgesia, opioids have a diminished efficacy as a result of receptor downregulation. Exaggerated pain responses are also apparent due to heightened NMDA receptor activity and neuroinflammation. Therefore, standard opioid dosing regimens may be insufficient for these patients, suggesting the need for high doses to counteract these effects. However, simply escalating dosing can increase withdrawal risks and contribute to OIH [4]. Providers must take a balanced approach to treating these patients to avoid negative implications such as hyperalgesia, respiratory depression, or prolonged recovery times. One possible solution is the implementation of multimodal analgesia and opioid rotation to optimize pain control while reducing dependence [10]. Another widespread approach is preoperative tapering of opioids to decrease the risk of hyperalgesia and lessen overall opioid consumption [13].

Effective perioperative pain management strategies for OUD patients all employ multimodal analgesia. Regional anesthesia, such as epidurals or nerve blocks, can be utilized to bypass opioid-mediated spinal sensitization [10]. The implementation of NMDA receptor blockades with NMDA antagonists like ketamine or dextromethorphan can reduce or reverse hyperalgesia [2]. Non opioid adjuncts (NSAIDs, gabapentinoids, acetaminophen) are capable of minimizing opioid reliance [14]. Another promising strategy is the continuation of opioid agonist therapy, such as methadone or buprenorphine, throughout the perioperative period to stabilize pain control and prevent withdrawal [8]. Ultimately, multimodal analgesia allows providers to properly enhance pain relief without heightening opioid-related complications.

Pharmacology management of OUD in the perioperative period

Managing perioperative pain in patients with OUD must consist of a balance between adequate analgesia, withdrawal prevention, and relapse risk mitigation. As discussed in the previous section, there are numerous neurobiological changes that take place in these patients that alter typical opioid pharmacodynamics, creating tolerance and OIH. Negative stigma surrounding this disorder, along with provider biases, creates another obstacle in proper treatment decisions. By incorporating evidence-based approaches, this section will serve to carefully examine the effectiveness of medications for opioid use disorder, opioid and non-opioid adjuncts, and multimodal strategies in improving outcomes. 

Methadone and Buprenorphine

Evidence suggests continuation of medications for OUD (methadone or buprenorphine) in the perioperative period may hold significant benefits. However, clinical guidelines regarding these treatments remain inconsistent and sparse [8].

Methadone shows clinical promise in diminishing the effects of opioid abuse during the surgical period. It is a full mu-opioid receptor agonist with a long half-life and NMDA receptor antagonism, allowing it to provide adequate analgesia and protection against OIH [12]. The general consensus for methadone advocates for continuation through the perioperative period, as abrupt cessation of this medication can lead to withdrawal and uncontrollable pain [15]. One study completed in 2020 suggests continuing oral intake of the medication on the day of the surgery; if oral intake is not feasible, IV methadone can be given at 50-75% of the daily dose, divided throughout 8-12 hours [2]. Specific clinical guidelines for continuation vs. temporary cessation of methadone are scarce, explaining the widespread variation in opinion among providers. Despite showing promising benefits, methadone does carry some risks as well. It prolongs the QT interval, requiring careful cardiac monitoring when given in high doses or when given to patients with cardiac risk factors [10]. 

Another common medication for OUD is buprenorphine, a partial MOR agonist with high receptor affinity and slow dissociation rates [8]. This strong receptor affinity can interfere with the analgesic efficacy of full agonist opioids. Buprenorphine is also a KOR antagonist and a delta-opioid receptor agonist. Just like methadone, there are conflicting recommendations as to whether providers should continue or temporarily cease buprenorphine administration before surgery. Some guidelines recommend continuing a low dose of buprenorphine perioperatively (≤8 mg/day) with an increased dosing frequency to improve analgesia, while others suggest transitioning to methadone or full MOR agonists 3-5 days before the procedure [5,12]. Many providers are weary about continuing administration through the perioperative period, as they fear that patients will require higher doses of full agonist opioids to receive effective analgesia. However, novel studies show that continuing buprenorphine, if in smaller, divided doses, can still allow for adequate pain control when multimodal analgesia strategies are utilized [2]. Non-opioid analgesics such as ketamine, NSAIDs, acetaminophen, and gabapentinoids are commonly used adjuncts. 

Full Opioid Agonists

Administering full opioid agonists presents many challenges for patients with OUD. These patients have increased tolerance, which heavily reduces analgesic efficacy, requiring the need for higher doses to achieve the same effect. Additionally, OIH exacerbates pain sensitivity as doses are escalated to meet pain control needs. Not only does perioperative opioid exposure heighten nociception and tolerance in these patients, but it also elevates relapse risk and can lead to opioid-seeking behaviors [11]. 

In order to prevent negative patient implications when using opioids, providers can take a few different approaches. Patient-controlled analgesia when using full agonists (morphine, hydromorphone, fentanyl) is one possible strategy, which allows individual titration to reach an adequate level of pain suppression [5]. Additionally, if higher doses are required in a patient, providers must employ multimodal strategies to prevent excessive opioid consumption and contribute to the development of OIH in patients. Another way to circumvent prolonged exposure risks is through the administration of shorter-acting opioids, which have less profound effects and provide a ceiling for respiratory depression.

Preventing Withdrawal and Relapse Postoperatively

Following a procedure, OUD patients face a serious risk of withdrawal and relapse, and providers must focus on creating standardized guidelines that mitigate these occurrences. Untreated withdrawal symptoms can cause a number of issues, including heightened pain levels, agitation, and potential relapse. To prevent this, patients who are on medications such as methadone or buprenorphine should promptly resume their regimens postoperatively [2]. Another beneficial strategy is the utilization of alpha-2 agonists like clonidine or dexmedetomidine, medications that suppress sympathetic activity by inhibiting central norepinephrine release, relieving patients of common withdrawal symptoms like anxiety, sweating, and tachycardia [5]. Providers should closely coordinate with addiction medicine specialists after the procedure to ensure the patient has a positive outcome after the patient’s recovery. Safe opioid tapering postoperatively allows patients to avoid prolonged exposure and relapse risks [12].

Multimodal analgesia

Multimodal analgesia (MA) is defined as the use of multiple agents and techniques targeting variable pain pathways to achieve greater pain relief, decrease opioid usage, and reduce side effects. MA is a critical treatment component for patients with OUD due to opioid tolerance, risk of relapse, and receptor differentiation. Opioid-induced hyperalgesia and tolerance render traditional opioid pain management regimens ineffective and risky. Additionally, providers must effectively work around the danger of central sensitization and reduced opioid efficacy that results from prolonged exposure. Modulating multiple pain pathways is one of the most effective ways to combat this challenge [7,14]. MA is a foundational strategy for postoperative pain in opioid-tolerant patients by enhancing analgesic efficacy while reducing opioid intake.

Key Components of Multimodal Analgesia

Each component of MA targets a distinct mechanism of pain signaling. Non-opioid systemic analgesics are a necessity in MA regimens due to their role as opioid-sparing agents. NSAIDs and acetaminophen decrease pain signaling at a molecular level by interfering with COX-1 and COX-2 enzymes, inhibiting prostaglandin synthesis [10,16]. With a decreased level of these hyperalgesic mediators, NSAIDs and acetaminophen also successfully reduce central sensitization. Another type of non-opioid systemic analgesic used in multimodal analgesia is gabapentinoids. Gabapentin, pregabalin, and other medications that fall under this class reduce excitatory neurotransmitter release, serving to be particularly helpful in neuropathic or opioid-tolerant states [14].

Alpha-2 adrenergic agonists, such as clonidine and dexmedetomidine, are also used in multimodal analgesia and serve a different purpose than NSAIDs. These drugs diminish central sympathetic outflow by inhibiting norepinephrine release, leading to additional benefits such as withdrawal management. Withdrawal symptoms are amplified by a mass release of norepinephrine in the CNS, demonstrating the effectiveness of alpha-2 agonists in combating this side effect. Additionally, these drugs can dampen pain perception by binding to alpha-2 receptors in the spinal cord, reducing the release of substance P and other nociceptive neurotransmitters. Ultimately, alpha-2 agonists serve a dual purpose of modulating pain pathways and inhibiting the presentation of withdrawal symptoms in OUD patients [5,8]. 

Ketamine, an NMDA receptor antagonist, counteracts central sensitization and pain amplification. Through the reduction of NMDA receptor signaling, ketamine decreases the excitability of neurons in the spinal cord, leading to decreased glutamate levels and diminished pain sensitivity. This NMDA antagonism marks ketamine as a crucial component of any multimodal analgesia regimen, as it can reduce OIH and other negative implications of chronic opioid use [2,7].

Peripheral nerve blocks are another vital tool in reducing systemic opioid usage. By directly targeting pain signaling at the spinal cord, these methods enhance pain control and provide other benefits over general anesthesia, such as reduced risks of pulmonary complications and faster recovery times. Regional approaches are a critical aspect of MA as they reduce postoperative pain and opioid consumption [13,14].

Multimodal Analgesia Effectiveness in OUD Populations

Significant evidence exists demonstrating the improved patient outcomes resulting from MA regimens tailored to OUD patients. This approach is integral to safe care in this population, as it reduces the risk of relapse and opioid consumption. Additionally, when supplemented with other forms of treatment for OUD patients, MA becomes even more effective. Numerous studies exist that report improved pain control when patients are maintained on opioid agonists such as buprenorphine or methadone [2,8]. Not only does this strategy allow providers to have more power over pain control, but it also allows patients to minimize opioid dosage escalation and avoid relapse triggers. To properly combat the spreading opioid crisis, providers must learn how to integrate non-opioid strategies that allow for decreased dosing and quicker recovery [9]. Though there is progress being made in the development and standardization of multimodal analgesia practices, challenges do remain. This approach requires careful coordination between all specialists working on a patient to avoid drug interactions and duplication of dosing. There is widespread variability in practice patterns of MA and a lack of standardized guidelines, serving as a call for uniform perioperative pain management strategies [12]. 

This individualized, multimodal strategy must be integrated into a treatment plan that is stigma-free and has structured discharge planning - two essential components to preventing relapse and aiding recovery.

Emerging Analgesic Therapies: Journavx

A promising advancement is the recent Food and Drug Administration (FDA) approval of Journavx (suzetrigine): a novel, non-opioid analgesic. Journavx is a first-in-class selective NaV1.8 sodium channel blocker that inhibits peripheral pain signal transmission without affecting the central nervous system, reducing the risk of dependence and misuse [17]. Approved in January 2025 for short-term use in adults with moderate to severe acute pain, it represents a significant step forward in non-opioid analgesia. In Phase 3 clinical trials, Journavx demonstrated efficacy comparable to opioids in postoperative settings for various procedures such as abdominoplasty and bunionectomy, and its utility is currently being evaluated in broader surgical contexts through ongoing Phase 4 studies. Unlike opioids, Journavx does not produce euphoric effects or respiratory depression, making it particularly valuable for OUD patients. It is administered orally with a 100 mg loading dose followed by 50 mg every 12 hours for a duration not exceeding 14 days. The most common side effects include pruritus, muscle spasms, elevated creatine phosphokinase, and rash [18]. Importantly, it is contraindicated with strong CYP3A inhibitors and grapefruit products due to metabolic interactions. The peripheral mechanism, strong analgesic potential, and favorable safety profile make Journavx a compelling adjunct or alternative to traditional multimodal analgesia strategies for this high-risk population.

Stigma and barriers to optimal treatment

Negative stigma towards patients with OUD is a significant barrier to providing compassionate, comprehensive care for this population. Implicit biases can take shape in undertreatment of pain, inadequate management of withdrawal symptoms, and even medical avoidance due to the patient’s fear of mistreatment. To properly address these issues, there needs to be OUD education, system-level reform, and a shift in the medical field so OUD is viewed as a chronic, treatable condition.

Impact of Stigma Among Healthcare Providers

The pervasiveness of implicit bias among healthcare workers contributes to the widespread under-treatment of patients with OUD. Health professionals may view these patients as drug-seeking or manipulative when providing care, often leading to withholding of necessary pain management solutions. Studies that have systematically reviewed attitudes of healthcare workers have discovered extensive negative perceptions towards these patients, specifically regarding trustworthiness and likelihood of treatment success [11]. Along with these negative beliefs, biases also take shape in prescribing patterns and overall care quality. There is a common clinical reluctance seen among providers that leads to an overreliance on subjective assessments when determining proper analgesia for OUD patients. Even in the face of clear clinical indications, health professionals have been reluctant to prescribe opioids and may inadequately dose analgesics due to fears of patient misuse or personal discomfort [5]. Not only can this stigma and bias lead to suboptimal prescribing, but it can also discourage the use of maintenance therapies (like buprenorphine or methadone) during the perioperative period. Opioid agonist therapy is a crucial aspect of perioperative treatment of OUD patients, and is often discontinued due to a lack of education on this disorder and negative beliefs towards these patients, worsening pain, and increasing risk of relapse [12].

Implicit bias and stigma manifest in inadequate pain management, which can have many consequences for these patients. OUD patients already face a high risk of developing OIH, and a lack of proper pain control only exacerbates the negative effects of this condition. Chronic opioid exposure alters pain processing, making conventional pain control strategies less effective [7]. Therefore, providers must closely listen and understand their patients to create the most beneficial treatment plan that avoids heightened pain perception. Along with worsening OIH, negative stigma can reduce patient engagement with the healthcare system altogether, impairing patient outcomes. This lack of engagement typically stems from patients refraining from disclosing their OUD and declining necessary procedures due to fears of being stigmatized and/or inadequately treated [11].

Patient Concerns and Psychological Barriers

In order to make progress in the treatment of this disorder, providers and the healthcare system as a whole must place more value on the patient and their feelings. To start, studies have shown that patients may fear withdrawal symptoms more than the pain surrounding their procedure. The physical and psychological distress often drives a higher anxiety response than the actual pain itself [15]. Therefore, providers and clinical guidelines should focus on the importance of treatments that proactively prevent withdrawal symptoms during the perioperative period, such as medications like alpha-2 agonists. These protocols should also place emphasis on providers establishing trust and rapport early in their relationship with the patient, as this is key to effective pain management in this population. If there is no solid provider-patient relationship, the patient may avoid disclosing substance abuse history and other vital information that is crucial for a proper treatment plan, hindering optimal care.

Reducing Stigma and Improving Care 

Improving the treatment of this underserved and neglected patient population necessitates widespread system changes. Provider education and institutional training on the neurobiology of addiction and evidence-based treatment strategies can significantly reduce bias and improve outcomes. Numerous new studies are advocating for the integration of addiction science into routine medical and clinical training in order to shift attitudes away from the existing negative biases [19]. Another beneficial change would be to adopt a chronic disease model for OUD, emphasizing it as a medical condition-like diabetes or hypertension-encouraging long-term management as opposed to the current dismissive attitudes that exist. Novel research describes OUD as a brain disease with well-established treatments in an attempt to urge the healthcare industry to align pain and addiction care adequately [9]. Additionally, addiction medicine should always be involved in perioperative planning with these patients in order to ensure continuity of care and recovery after a surgical admission. Multidisciplinary coordination is essential when dealing with complex decisions for the patient, such as opioid tapering or transitioning to other therapies [12]. Lastly, clear protocols for the management of patients on opioid agonist therapies must be established; guidelines can minimize provider uncertainty and reduce subjective decision-making. Clear instructions on how to continue opioid agonist therapy while layering this treatment with multimodal analgesia will provide the patient with safe, effective pain relief while simultaneously reducing stigma and relapse risk.

Addressing and mitigating stigma among providers is one of the first steps to improving care for OUD patients. However, lasting progress can only be made through the creation and implementation of evidence-based clinical guidelines. There is a critical need for standardized protocols and continued research in order to combat the growing complexity of this disease.

Clinical guidelines and future research

Despite mounting evidence supporting specific perioperative strategies regarding the management of OUD, there is still widespread variability in the clinical treatment of these patients. Numerous topics, including the continuation of opioid agonist therapy, multimodal analgesia, and inadequate provider education, have limited guidelines, which have resulted in suboptimal outcomes. The complexity of treating OUD, which is enhanced by opioid-induced hyperalgesia, tolerance, and relapse risk, highlights the need for clear clinical protocols. This section demonstrates the need for standardized, multidisciplinary approaches and outlines key areas of research that can improve outcomes.

Current Recommendations and Institutional Protocols

A significant issue with current approaches to treating this disorder lies in the fact that existing guidelines vary by institution and speciality, creating inconsistent methods to manage OUD perioperatively. The American Society of Anesthesiologists (ASA) and American Society of Regional Anesthesia and Pain Medicine (ASRA) have issued general recommendations, but for certain topics, such as opioid agonist therapy continuation, these instructions lack specific guidance [8]. Many institutions' current guidelines suggest continuing opioid agonist therapy during surgery with supplemental full opioid agonists and multimodal analgesia [2,12]. Additionally, institutions generally recommend regional anesthesia, ketamine infusion, and alpha-2 agonists as adjuncts for improving pain control without increasing the risk of relapse [4]. Though there is a general lack of protocol, most institutions and societies agree on the need to manage patients in a way that prevents withdrawal and maintains stability for patients to continue on opioid agonist therapy [8].

Need for Standardized Guidelines

The existing lack of national consensus on proper perioperative treatment leaves anesthesiologists and surgeons uncertain about optimal strategies. Studies have shown that variability in practice leads to poor patient outcomes and an increased risk of relapse and undertreated pain in this population [7,19]. One especially inconsistent area is buprenorphine management, as some institutions recommend continuing it perioperatively, and others advocate for tapering before the procedure and switching to full agonists [2,8]. Another shortcoming of the current system is the absence of multidisciplinary care models. To properly treat a disorder as convoluted as OUD, coordination between anesthesia, pain specialists, addiction medicine, and primary care is required. These models must emphasize individualized approaches based on the type of surgery, the patient’s condition, and access to postoperative support [13]. 

Areas for Future Research

Multiple aspects of proper OUD treatment are severely understudied, and future research can bring solutions to these issues and optimize patient outcomes. For example, the long-term effects of different opioid agonist therapies are poorly researched. Randomized, controlled trials comparing continuation vs. cessation of buprenorphine or methadone in elective surgeries will be crucial in determining the most beneficial perioperative strategy. When it comes to multimodal analgesia, current data support reduced opioid use and better patient outcomes, but there are a limited number of studies that focus solely on OUD patients [10,16]. If further research were performed, clinical guidelines on the ideal combinations of non-opioid adjuncts could be created. Another area of research to investigate is how educational and policy changes can improve provider attitudes and patient outcomes. Lastly, the creation of a national database that could help track trends, complications, and effective interventions across institutions would be profoundly beneficial [9]. As the perioperative management of OUD patients continues to evolve, it is important to bridge the gaps in current research and the lack of clinical guidelines. In order to do this, evidence-based protocols must be created and supported by a multidisciplinary approach and increased provider education to improve patient outcomes in this population. With this foundation in place, this review will now focus on broader implications and future goals.

## Conclusions

As the number of patients with OUD undergoing surgery continues to rise, institutions must construct a patient-centered approach to this issue. Traditional opioid dosing strategies present numerous limitations when treating this population, necessitating novel and unique clinical strategies that include multimodal analgesia, continuity of opioid agonist therapy, and withdrawal/relapse prevention. Apart from pharmacological changes that must be implemented, decreasing the stigma surrounding these patients and improving interdisciplinary coordination between health professionals are crucial. Establishing standardized clinical guidelines will create a pivotal foundation for advancing perioperative care outcomes for patients with opioid use disorder.
